# Are patient factors associated with real-world antibody–drug conjugate outcomes in gynecologic cancers?

**DOI:** 10.1016/j.ygyno.2026.04.011

**Published:** 2026-04-28

**Authors:** Eliya K. Shachar, Jordyn Silverstein, Lorna Kwan, Ritu Salani, Beth Y. Karlan, Dana M. Chase

**Affiliations:** a Department of Obstetrics and Gynecology, University of California, Los Angeles, CA, USA; b Gray Faculty of Medicine, Tel Aviv University, Tel Aviv, Israel; c Division of Hematology/Oncology, Department of Medicine, University of California, Los Angeles, CA, USA; d Department of Urology, University of California, Los Angeles, CA, USA

**Keywords:** Antibody drug conjugates, Gynecologic cancer, Race, Ethnicity, Age, Underrepresented minorities

## Abstract

**Objectives.:**

Disparities in clinical trial enrollment raise concerns about the generalizability of antibody–drug conjugate (ADC) efficacy in gynecologic cancers. We evaluated real-world survival outcomes across demographic subgroups and assessed concordance with pivotal trials, while contextualizing findings within ongoing challenges of underrepresentation.

**Methods.:**

We conducted a cohort study of patients with advanced gynecologic cancers treated with ADCs at a tertiary academic center (June 2019–September 2025). Primary outcomes were overall survival (OS) and progression-free survival (PFS) by age, race, and ethnicity. Secondary objectives examined ECOG performance status, line of therapy, and Area Deprivation Index. Exploratory analyses assessed treatment discontinuation secondary to toxicity. Survival was estimated using Kaplan–Meier methods with univariable and multivariable models.

**Results.:**

Among 142 patients,105 received mirvetuximab soravtansine (MIRV), 34 trastuzumab deruxtecan (T-DXd), and 16 tisotumab vedotin (TV). Median age was 64.8 years; 66.9% identified as White, 12.7% Asian, 6.3% Black/African American, and 14.1% Other. Most patients were non-Hispanic/Latina (88.7%), while 11.3% identified as Hispanic/Latina. In unadjusted analyses, PFS varied by treatment line in the MIRV cohort (*p* = 0.04), while OS differed by ethnicity and performance status (*p* < 0.01). Performance status was also associated with OS in the T-DXd cohort (*p* = 0.01). These associations were not significant in multivariable models. Treatment discontinuation due to toxicity occurred in 17.6% and did not differ by subgroup.

**Conclusions.:**

Real-world ADC outcomes were consistent with pivotal trials, with no independent survival differences by subgroup after adjustment. These findings support continued investigation of clinical and structural factors influencing outcomes.

## 1. Introduction

Antibody drug conjugates (ADCs) combine the tumor-targeting specificity of monoclonal antibodies with the cytotoxic potency of linked chemotherapeutic payloads and have recently transformed treatment paradigms in advanced ovarian, cervical, and endometrial cancers. FDA-approved ADCs in gynecologic oncology include mirvetuximab soravtansine (MIRV) for folate receptor-α–expressing platinum-resistant ovarian cancer, trastuzumab deruxtecan (T-DXd) for HER2-expressing uterine, ovarian, and cervical cancers, and tisotumab vedotin (TV) in recurrent or previously treated metastatic cervical cancer [1–6]. Additional ADCs targeting novel antigens with varied payloads are under active development and clinical investigation [7].

Despite these therapeutic advances, racial and ethnic populations and older adults remain persistently underrepresented in oncology clinical trials, even in the setting of the National Institutes of Health mandates promoting inclusion [8,9]. Among oncology trials leading to FDA drug approvals between 2008 and 2018, Black patients comprised only 3.1% and Hispanic patients 6.1% of participants—representing just 22% and 44% of expected enrollment based on U.S. cancer incidence, respectively [10].

Trial eligibility criteria frequently exclude patients with heavily pretreated disease or poor performance status, thereby limiting generalizability to real-world populations. Moreover, most clinical trials do not incorporate broader social determinants of health, such as the Area Deprivation Index (ADI)—a composite, neighborhood-level measure of socioeconomic disadvantage encompassing income, education, employment, and housing quality—which has been consistently associated with inferior cancer outcomes across multiple malignancies [11–13]. A study using data from the Texas Cancer Registry investigated how ADI mediates racial and ethnic disparities in diagnosis of late-stage cervical cancer [14]. Non-Hispanic Black and Hispanic/Latina individuals had higher ADI scores and greater odds of late-stage diagnosis compared with non-Hispanic White individuals, suggesting that neighborhood-level social determinants substantially mediate these disparities.

Racial, ethnic and age disparities in trial enrollment may limit the generalizability of treatment outcomes for emerging cancer therapies, including ADCs used in gynecologic malignancies. Limited representation of these populations raises concerns that the efficacy and toxicity profiles derived from pivotal ADC trials, which predominantly enrolled White and younger patient populations, may not fully reflect outcomes in real-world settings. Diverse populations often present with higher comorbidity burdens, heterogeneous tumor biology, and varied social and structural determinants of health, all of which may influence treatment response and survival [15].

Previous literature reveals substantial racial and ethnic disparities in access and outcomes with ADCs in breast cancer, driven by structural determinants of health, including insurance, socioeconomic factors, and barriers to care [16]. Real-world studies in HER2-positive metastatic breast cancer have shown significantly worse survival outcomes for Black patients compared with Asian, Hispanic, and non-Hispanic White patients, with disparities attributed to multi-level structural and clinical factors [16]. Additional institutional and real-world analyses of sacituzumab govitecan report higher rates of disease progression among Black/African American patients [17].

Due to the concern that underrepresented groups may have worse outcomes, the primary objective of this study was to evaluate real-world treatment outcomes of ADC therapy in patients with advanced gynecologic cancers, with a focus on historically underrepresented racial/ethnic groups and older adults, and to assess whether overall survival (OS) and progression-free survival (PFS) are concordant with outcomes reported in clinical trials.

## 2. Methods

### 2.1. Study design and population

We conducted a single-institution, retrospective cohort study of patients ≥18 years with metastatic or recurrent gynecologic cancers (ovarian, endometrial, or cervical) treated with an FDA-approved ADC between June 2019 and September 2025 at University of California, Los Angeles. Patients were identified through the institutional electronic health record (EHR) system. The study was approved by the institutional review board (IRB-24–6039).

### 2.2. Eligibility criteria

Eligible patients were ≥18 years old at gynecologic cancer diagnosis, had a pathologically confirmed gynecologic malignancy, and received at least one dose of an ADC, MIRV, T-DXd, or TV. Eligible cases required available demographic and clinical data within the EHR. Patients were excluded if they were <18 years or lacked sufficient demographic or clinical information.

### 2.3. Data collection

Demographic variables included age, race, ethnicity, and socioeconomic status measured by ADI. Clinical variables included primary cancer site, histology, Eastern Cooperative Oncology Group (ECOG) performance status, prior lines of therapy, and treatment-related toxicities resulting in treatment discontinuation [18,19].

Socioeconomic status was measured using the ADI from the University of Wisconsin Neighborhood Atlas [20,21], which ranks census block groups at both national and state levels, with higher values indicating greater socioeconomic disadvantage. Residential consensus block groups were linked to corresponding ADI percentiles. For primary analyses, ADI was dichotomized using an empirically supported threshold within our cohort to define low deprivation (≤9) and high deprivation (>9), consistent with prior literature; sensitivity analyses applied quartile-based categorizations. Because national and state ADI ranks were highly correlated (*r* = 0.84), all analyses were conducted and reported using national ADI rankings.

### 2.4. Outcome measures

Outcomes included PFS, defined as months from ADC initiation to documented disease progression or death from any cause, whichever occured first, and OS, defined as months from ADC initiation to death from any cause.

Secondary objectives evaluated associations between OS and PFS with line of therapy, performance status, and socioeconomic context as measured by the ADI. Exploratory analyses assessed discontinuation secondary to treatment toxicity by age, race and ethnicity.

### 2.5. Statistical analysis

Descriptive statistics summarized demographic and clinical characteristics. Categorical variables were compared using Chi-square (or Fisher's exact) tests. PFS and OS for each ADC were estimated using Kaplan–Meier methods, with primary objective evaluating subgroups by race, ethnicity and age categories performed using log-rank tests. Secondary objectives assessed PFS and OS by ECOG performance status (0–1 vs. 2–4), line of treatment (1–2 vs. ≥3), and ADI (low vs. high; quartile-based sensitivity analyses).

To identify independent predictors of PFS and OS, multivariable Cox proportional hazards regression models were constructed separately for each ADC cohort (MIRV, T-DXd, and TV). Covariates were selected a priori based on clinical relevance. The following variables were included in all models: age at ADC treatment initiation, race, ethnicity, ECOG performance status at ADC initiation (included only in the MIRV cohort, as the T-DXd and TV cohorts were limited by small sample size), line of therapy, treatment discontinuation due to toxicity, and biomarker status.

Biomarker status was incorporated based on specific disease site. For the ovarian cancer MIRV cohort, biomarkers included FRα expression (high ≥75% vs low/intermediate <75%), BRCA mutation status (germline or somatic), homologous recombination repair status, and loss of heterozygosity. In the T-DXd cohort, HER2 status was evaluated using ERBB2 amplification, combined IHC expression and amplification, and an ordinal IHC scale (0, 1+, 2+, 3+). For the cervical patients treated with TV, PD-L1 expression was categorized using the Combined Positive Score (CPS ≥1 vs <1).

Hazard ratios (HRs) with 95% confidence intervals (CIs) were estimated for each covariate. All statistical tests were two-sided, with *p* < 0.05 considered statistically significant. Analyses were performed using SAS 9.4 (Cary, NC).

## 3. Results

### 3.1. Patient characteristics

A total of 142 patients with gynecologic malignancies received ADCs, including 105 (73.9%) treated with MIRV, 34 (23.9%) with T-DXd, and 16 (11.3%) with TV (Table 1). The median age at ADC initiation was 64.8 years (IQR, 56.5–73.5), with cohort-specific medians ranging from 55.7 years in the TV cohort to 64.6 years among MIRV-treated patients and 67.7 years in those receiving T-DXd. Two-thirds of patients identified as White (66.9%), with additional representation from Asian (12.7%), Black/African American (6.3%), and Other racial groups (14.1%). The majority identified as non-Hispanic/Latina (88.7%), while 11.3% identified as Hispanic/Latina ethnicity.

Performance status at ADC initiation was generally favorable: 60.9% of patients had ECOG 0 and 31.9% had ECOG 1, while 7.3% had poor performance status (ECOG ≥2). Only one patient had an ECOG of 3. The proportion of patients with ECOG ≥2 was 5.9% in the MIRV cohort, 8.8% among those receiving T-DXd, and 6.2% in the TV cohort. Among patients with ECOG ≥2 (*n* = 10), 60% were non-Hispanic/Latina, 20% Hispanic/Latina, and 20% of Other ethnicity; by race, 50% were White, 30% Other, 10% Asian, and 10% Black/African American. Overall, baseline ECOG performance status, categorized as good (0–1) versus poor (≥2), did not differ significantly by race or ethnicity across the ADC-treated population (*p* > 0.05).

Ovarian and uterine cancers accounted for the largest proportion of diagnoses, with serous histology representing 82.0% of cases. Among cervical cancer patients treated with TV, the distribution of histologic subtypes included squamous cell carcinoma (43.8%), adenocarcinoma (43.8%), and adenosquamous carcinoma (12.5%).

Patients were heavily pretreated, initiating ADC therapy at a median of fourth-line treatment (range, 2–10). The median number of ADC cycles administered was six overall, with the longest exposure observed in the MIRV cohort (median, 7 cycles) and the shortest in the TV cohort (median, 3 cycles).

Across MIRV- and T-DXd–treated patients, 17.3% (*n* = 24) received ADCs in combination with another agent, including bevacizumab, immune checkpoint inhibitors, carboplatin, or gemcitabine at the clinician's discretion (Supplementary Table S1). Of the patients treated with combination-regimens, 91.7% were non-Hispanic/Latina and 8.3% other ethnicity; racially, 66.7% were White, 12.5% Asian, 8.3% Other, and 4.2% Black/African American.

### 3.2. Overall survival outcomes by race, ethnicity and age

Among patients treated with **MIRV**, median OS was 21.3 months (95% CI, 14.4–33.8) and was comparable between those age ≥65 years (21.3 months, 95% CI, 13.0–33.8) and <65 years (21.8 months, 95% CI, 12.4–not estimable (NE); *p* = 0.84, Table 2, Supplementary Fig. S1). Survival by race was numerically longer in White patients (21.3 months, 95% CI, 15.5–NE) compared with non-White patients (14.4 months, 95% CI, 11.5–NE; *p* = 0.81). Ethnicity-based analyses revealed significant differences with non-Hispanic patients having a median OS of 24.4 months (95% CI, 16.9–35.8), Hispanic patients 11.8 months (95% CI, 5.2–43.2), and patients of Other ethnic backgrounds 3.4 months (95% CI, 1.4–11.5; *p* < 0.0001) (see Table 3.)

In the **T-DXd-treated cohort**, median OS was 12.0 months (95% CI, 7.8-NE), and did not differ significantly by age (≥65: 13.0 months, 95% CI, 7.8, NE vs <65: 11.3 months; 95% CI, 2.4–NE; *p* = 0.31), race (White: 12.1 months, 95% CI, 7.1–NE vs non-White: 13.0 months, 95% CI, 4.8–NE; *p* = 0.52), or ethnicity (Hispanic: 14.2 months, 95% CI, NE vs non-Hispanic: 12.0 months, 95% CI 12.0 months, 95% CI 7.8-NE; *p* = 0.97, Supplemental Fig. S2).

The median OS of patients treated with TV was 9.0 months (95% CI, 3.3–21.0), and did not significantly differ across age groups (≥55: 9.0 months; 95% CI, 3.3–36.8 vs <55: 7.4 months; 95% CI, 1.7–21.0; *p* = 0.36; Supplemental Fig. S3). Race-based comparisons showed a numerical difference in median OS of 21.0 months (95% CI, 3.3–36.8) for White patients and 5.6 months (95% CI, 1.7–11.3) in non-White patients (*p* = 0.13). By ethnicity, OS was 6.5 months (95% CI, 1.7–11.3) among Hispanic patients versus 9.0 months (95% CI, 3.3–36.8) for non-Hispanic patients (*p* = 0.26).

### 3.3. Overall survival outcomes by treatment line, performance status and ADI

OS did not differ by prior lines of treatment in patients treated with any of the ADCs. In contrast, ECOG performance status was strongly associated with survival outcomes (*p* = 0.005): patients treated with MIRV and good performance status (ECOG 0–1, *n* = 93) had a median OS of 21.8 months, (95% CI, 16.3–35.8) compared to 8.8 months (95% CI, 1.2-NE) in the poor performance status subgroup (ECOG ≥2, *n* = 6). Similarly, OS of patients treated with T-DXd, was longer for patients with good performance status (ECOG 0–1, *n* = 31; median 13.0 months, 95% CI, 10.4-NE) compared to those with less favorable performance status (ECOG ≥2, *n* = 3; median 4.8 months, 95% CI, 0.1–4.8; *p* = 0.001). In contrast, ECOG performance status was not significantly associated with OS in the TV-treated cohort (*p* = 0.76). Median OS did not vary by ADI in any of the three ADC treatment groups.

### 3.4. Progression-free survival among underrepresented populations

Across ADC cohorts, PFS generally mirrored OS findings, with limited associations by age, race, or ethnicity. Among MIRV-treated patients, median PFS was 7.3 months (95% CI, 5.5–13.5), and did not differ by age, race or ethnicity; however, in unadjusted analyses, PFS varied by line of treatment, with shorter PFS among greater than 3rd line of treatment (6.63 months; 95% CI, 4.7–9.4) compared with 1st or 2nd line of treatment (16.8 months; 95% CI, 5.5-NE) (*p* = 0.04; Supplemental Fig. S4). ECOG performance status and ADI were not significantly associated with PFS in the MIRV cohort.

In the T-DXd-treated population, median PFS was 6.5 months (95% CI, 2.4-NE), and similar across age, race, ethnicity, line of therapy, performance status and ADI. Among TV-treated patients, PFS did not differ by demographic or clinical factors, with the exception of trend in socioeconomic status: patients residing in low-ADI areas experienced numerically longer PFS (median, NE months; 95% CI, 1.4-NE) compared with those in high-ADI areas (median, 3.2 months; 95% CI, 1.4-NE; *p* = 0.05).

### 3.5. Treatment discontinuation by race, ethnicity and age

Treatment discontinuation due to toxicity occurred in 17.6% (*n* = 25) of the overall cohort, including 15.2% (*n* = 16) of MIRV-treated patients, 14.7% (*n* = 5) of those receiving T-DXd, and 25.0% (*n* = 4) of the TV cohort. Reported toxicities leading to discontinuation included pneumonitis (*n* = 5, 4.8%), including one fatal grade 5 event; ocular toxicity (*n* = 6, 5.7%); neuropathy (*n* = 1, 1.0%); hematologic toxicity (*n* = 2, 1.9%); and unspecified toxicities (n = 2, 1.9%). In the T-DXd cohort, 5 (14.7%) patients discontinued treatment due to toxicity, secondary to pneumonitis (n = 1, 2.9%), cardiac toxicity with diarrhea (*n* = 1, 2.9%), severe emesis and abdominal pain (*n* = 1, 2.9%), and unspecified toxicities (*n* = 2, 5.9%). Among patients treated with TV, 4 patients (25.0%) discontinued therapy due to toxicity, including bleeding events (n = 2, 12.5%), such as gastric erosions and hematemesis; asthenia (n = 1, 6.3%); and small bowel perforation (n = 1, 6.3%).

In the overall ADC-treated cohort, those who discontinued therapy due to toxicity, 88% were non-Hispanic/Latina, 8% Hispanic/Latina, and 4% other ethnicity; racially, 64% were White, 24% Other, 8% Asian, and 4% Black/African American. Discontinuation rates were similar by age (<65 vs. ≥65 for MIRV and T-DXd, and <55 vs. ≥55 for TV; *p* = 0.56), race (White vs. non-White; *p* = 0.52), and ethnicity (Hispanic/Latino vs. non-Hispanic; *p* = 0.89). Overall, toxicity-related treatment cessation appeared consistent throughout subgroups.

### 3.6. Multivariate analysis

In multivariable Cox proportional hazards models evaluating OS and PFS across ADC-treated cohorts, race, ethnicity, age, line of treatment, ECOG performance status, socioeconomic status (ADI), biomarker status (therapy-dependent), and treatment discontinuation due to toxicity were not independently associated with survival outcomes across MIRV, T-DXd, and TV cohorts. ECOG and toxicity-related discontinuation variables were excluded from select ADC-specific models (T-DXd, and TV) due to limited event counts.

## 4. Discussion

In this real-world study of patients with advanced gynecologic malignancies treated with ADCs, survival outcomes varied by agent and clinical characteristics. However, demographic factors—including race, ethnicity, and age—were not independently associated with survival after adjustment.

Across ADCs, univariant analysis demonstrated that ECOG performance status is a prognostic determinant of survival, underscoring the central role of functional status in treatment selection and outcome estimation in contemporary oncology practice. These findings align with prior evidence indicating that functional status often outweighs chronological age in predicting benefit from novel targeted therapies.

In unadjusted analyses, differences in OS by ethnicity were observed in select cohorts, most notably among MIRV-treated patients; however, these associations were not sustained in multivariable models. This finding suggests that apparent disparities are more likely attributable to underlying clinical factors, particularly baseline performance status, rather than demographic characteristics themselves. Alternatively, limited cohort size may have constrained the ability to detect modest independent effects.

Observed ethnicity-based differences in unadjusted outcomes within the MIRV-treated population are unlikely to be explained by treatment discontinuation due to toxicity, as most patients who discontinued therapy were non-Hispanic and White. In contrast, non-White and Hispanic patients were proportionally overrepresented among those with poorer ECOG performance status at treatment initiation, a factor more directly associated with inferior outcomes across analyses.

Notably, line of therapy was significantly associated with PFS among patients treated with MIRV, suggesting that MIRV is most effective when introduced in earlier line, while patients treated with T-DXd and TV derived comparable benefit from treatment regardless of treatment line, suggesting that these ADCs retain therapeutic activity even in heavily pretreated populations.

The study cohort experienced higher rates of treatment discontinuation due to toxicities—comprised predominantly of pneumonitis and ocular adverse events—compared with rates reported in clinical trials. In trials, discontinuation rates were substantially lower: 9.2% in MIRASOL for MIRV, 7.5% for T-DXd in the DESTINY-PanTumor02 endometrial cohort and 2.5% in the ovarian cohort, and 14.8% in the innovaTV 301 trial for TV—all notably below the rates observed in our real-world population (17.6%) [1,3,4]. This increased discontinuation may reflect factors such as greater comorbidity burden, more heterogeneous pretreatment exposures, use of combination regimens, broader inclusion of patients with poorer performance status, and less stringent monitoring or dose-modification protocols relative to controlled trial settings.

## 5. ADC-specific findings

### 5.1. Mirvetuximab soravtansine

Median OS (21.3 months) and median PFS (7.3 months) in the MIRV-treated cohort exceeded outcomes reported in MIRASOL and SORAYA [2,3]. This difference may reflect real-world treatment patterns, including use of combination regimens, with 21.0% of patients who received MIRV off-label with bevacizumab, carboplatin, or gemcitabine. Notably, these favorable OS outcomes were observed despite the inclusion of patients with poorer performance status and those treated beyond third-line therapy. Interestingly, patients receiving MIRV in more advanced settings, derived less benefit as reflected in shorter PFS in the univariate analysis. While MIRASOL demonstrated consistent efficacy across age, variables such as race, ethnicity-specific outcomes were not reported, and eligibility was restricted to patients with ECOG 0–1. Our findings extend these data to a more heterogeneous population, while reinforcing that ethnicity and performance status was not an independent predictor of outcome after adjustment.

Moreover, among patients receiving combination ADC regimens with MIRV or T-DXd, the majority (91.7%) were non-Hispanic/Latina, introducing potential selection bias that may partially account for the numerically more favorable outcomes observed in this subgroup.

### 5.2. Trastuzumab deruxtecan

For patients treated with T-DXd, median OS was 12.0 months and median PFS 6.5 months, notably 17.6% of the cohort had +1 IHC expression suggesting efficacy in HER2 low expressing patients. Although limited by sample size, OS did not differ significantly across clinical and demographic subgroups, supporting the premise that clinical benefit from T-DXd is primarily driven by underlying tumor biology—specifically HER2 target expression—rather than sociodemographic characteristics.

Patients classified as Black or Other race uniformly met established T-DXd biomarker eligibility criteria, whereas White and Asian patients more frequently received T-DXd under less stringent criteria, such as IHC 1+ expression. This finding may reflect differences in biomarker treatment selection practices across racial groups.

### 5.3. Tisotumab vedotin

Survival outcomes with TV reported in innovaTV 301 trial (median OS, 11.5 months, and median PFS, 4.2 months) [6,22] varied slightly, with a median OS and PFS of 9.0 months and 4.8 months, respectively in our cohort. The shorter OS in our real-world cohort, may reflect the inclusion of more heavily pretreated patients and those with poorer baseline performance status, factors known to adversely influence outcomes in recurrent or metastatic disease. While PFS was similar to established TV efficacy as demonstrated in clinical trials. Although White, non-Hispanic patients and individuals residing in lower-ADI areas demonstrated numerically longer OS, these differences were not statistically significant and were likely limited by small sample size. Notably, among TV-treated patients, PFS was numerically longer for those residing in low-ADI areas (median, 11.2 months) compared with those in high-ADI areas (median, 2.45 months) in unadjusted analyses. However, this association was not retained in multivariable models, underscoring the need for confirmation in larger, independent cohorts.

## 6. Limitations

This study has several methodological limitations inherent to its retrospective, real-world design. A key limitation is the single-institution setting, as the insurance mix at UCLA may reflect a patient population with relatively greater socioeconomic resources compared with other care environments, potentially limiting generalizability to more socioeconomically disadvantaged populations. Modest sample sizes, particularly within agent-specific and demographic subgroups limited statistical power to detect subtle but potentially clinically meaningful differences. The cohort was predominantly White, with fewer patients from underrepresented racial and ethnic groups, reflecting persistent disparities in real-world access to novel therapies and clinical trial participation. This imbalance limits the precision of subgroup analyses and may obscure outcome variability in more diverse populations. Although the cohort primarily comprised ovarian and uterine cancers, a small number of cervical cancer cases were included to reflect real-world TV use; however, given the small sample size, these cases contribute minimally and do not support disease-specific conclusions.

Additionally, the inclusion of multiple gynecologic malignancies, heterogeneous prior treatments, and varying lines of therapy introduces potential confounding, as do unmeasured factors such as comorbidity burden, dose modifications, and differences in supportive care. Despite these limitations, the consistency of non-significant demographic variables across ADCs suggest that these factors likely play a less meaningful role in treatment outcome. Observed unadjusted differences in the subgroups warrant cautious interpretation because of limited subgroup size and statistical power.

## 7. Strengths

This work is novel in its evaluation of real-world survival outcomes across demographic, clinical, and socioeconomic subgroups that were not comprehensively examined in pivotal ADC trials. While MIRASOL reported subgroup analyses by age, the trials leading to FDA approval of T-DXd (DESTINY-PanTumor02) and TV (innovaTV 301) did not report survival outcomes stratified by age, race, or ethnicity. Moreover, this study extends existing evidence by including heavily pretreated patients and those with poorer performance status who were largely excluded from these trials. Notably, MIRASOL limited enrollment to patients with up to three prior lines of therapy, innovaTV 301 to one to two prior lines, whereas DESTINY-PanTumor02 allowed more extensive pretreatment. In addition, our analysis incorporates neighborhood-level socioeconomic context using ADI, further enhancing real-world generalizability.

## 8. Conclusions

In summary, real-world survival outcomes with ADCs in gynecologic cancers largely align with clinical trial experience, while extending existing evidence to more clinically heterogeneous and heavily pretreated populations that are frequently underrepresented in prospective studies. Age, race, ethnicity, ECOG performance status, line of therapy, ADI and toxicity were not independently associated with outcomes after adjustment.

As ADC utilization expands across gynecologic malignancies, improving the accrual of underrepresented populations in clinical trials remains critical. Future studies integrating social determinants of health with molecular and clinical factors will be essential to disentangle biological mechanisms from structural contributors to outcome variability and ensure equitable evaluation and delivery of emerging therapies.

## Supplementary Material

Supplementary Meterial

## Figures and Tables

**Table 1:** Gynecologic Cancer Patients treated with an Antibody Drug Conjugates.

Variable	Total Population* (*n* = 142) N (%)	Mirvetuximab soravtansine (*n* = 105)* n (%)	Trastuzumab deruxtecan (*n* = 34)* n (%)	Tisotumab vedotin (*n* = 16)* n (%)

**Age at ADC initiation (yrs),** Median (IQR)	64.8 (56.5–73.5)	64.6 (57.3–73.3)	67.7 (60.3–73.5)	55.7 (45.6–69.8)
**Race**				
White	95 (66.9)	72 (68.6)	22 (64.7)	10 (62.5)
Asian	18 (12.7)	12 (11.4)	4 (11.8)	3 (18.8)
Black/African American	9 (6.3%)	6 (5.7)	4 (11.8)	3 (18.8)
Other	20 (14.1)	15 (14.3)	4 (11.8)	0 (0)
**Ethnicity**				
Not Hispanic/Latino	126 (88.7)	96 (91.46)	31 (91.23)	11 (68.8)
Hispanic/Latino	16 (11.3)	9 (8.6)	3 (8.8)	5 (31.2)
**ECOG PS**				
0	84 (60.9)	614 (63.4)	17 (50.0)	12 (75.0)
1	44 (31.9)	31 (30.7)	14 (41.2)	3 (18.8)
≥2	10 (7.3)	6 (5.9)	3 (8.8)	1 (6.2)
**Primary Cancer Site, Histology**				
**Ovarian/UterineCancers** (*n* = 139)				
Serous	114 (82.0)	93 (88.6)	21 (61.8)	
Clear cell	6 (4.3)	3 (2.9)	3 (8.8)	
Carcinosarcoma	10 (7.2)	6 (5.7)	4 (11.8)	
Endometrioid	5 (3.6)	3 (2.9)	2 (5.9)	
Other	4 (2.9)	0	4 (11.8)	
**Cervix**				
Squamous cell carcinoma				7 (43.8)
Adenocarcinoma				7 (43.8)
Adenosquamous carcinoma				2 (12.5)
**HPV**				
HPV independent				3 (18.8)
HPV associated				9 (56.3)
Unknown				4 (25.0)
**CPS PDL-1**				
Positive ≥1				11 (68.8)
Negative <1				3 (18.8)
Unknown				2 (12.5)
**ADC Line of treatment,** median (range)	4 (2–10)	4 (2–10)	4 (2–10)	2 (2–8)
2	38 (24.5)	24 (23.1)	5 (15.6)	9 (56.2)
3	36 (23.2)	22 (21.2)	9 (28.1)	5 (31.2)
4	32 (20.6)	28 (26.9)	4 (12.5)	1 (6.2)
5	17 (11.0)	10 (9.6)	7 (21.9)	0 (0)
6	14 (9.0)	11 (10.6)	3 (9.4)	0 (0)
7	5 (3.2)	5 (4.8)	0 (0)	0 (0)
8	4 (2.6)	2 (1.9)	1 (3.1)	1 (6.2)
9	3 (1.9)	1 (1.9)	2 (6.3)	0 (0)
10	2 (1.3)	1 (1.0)	1 (3.1)	0 (0)
**ADC cycles,** median (range)	6 (1–48)	7 (1 –48)	6 (1–31)	3 (1–11)
**Adverse events leading to discontinuation**	25 (17.6)	16 (15.2)	5 (14.7)	4 (25.0)

* Some patients had synchronous primary tumors and were therefore represented in more than one ADC cohort. Percentages were rounded to one decimal place, and thus totals may not sum to exactly 100%.

**Table 2:** Median Overall Survival (months) for Gynecologic Antibody–Drug Conjugates by Age, Race, Ethnicity, Line of Treatment, ECOG Performance Status, and Area Deprivation **Index.**

Variable	MIRV			T-DXd			TV		
	Median OS	(95% CI)	*p*-value	Median OS	(95% CI)	p-value	Median OS	(95% CI)	p-value

**Total**	21.3	(14.4, 33.8)	–	12.0	(7.8, NE)	–	9.0	(3.3, 21.0)	–
**Age**									
<65/55	21.8	(12.4, NE)	0.84	11.28	(2.40, NE)	0.31	7.37	(1.74, 21.02)	0.36
≥55/65	21.3	(13.03, 33.75)		13.03	(7.83, NE)		9.05	(3.26, 36.81)	
**Race**									
White	21.3	(15.49, NE)	0.81	12.04	(7.07, NE)	0.52	21.02	(3.26, 36.81)	0.13
Non-White	14.44	(11.55, NE)		13.03	(4.84, NE)		5.58	(1.74, 11.32)	
**Ethnicity**									
Hispanic	**11.78**	**(5.16, 43.22)**	**<0.0001**	14.24	(NE)	0.97	6.51	(1.74, 11.32)	0.26
Non-Hispanic	**24.38**	**(16.88, 35.82)**		12.04	(7.83, NE)		9.05	(3.26, 36.81)	
Other	**3.37**	**(1.38, 11.55)**		NE			–		
**Treatment Line**									
1–2	NE	(11.28, NE)	0.42	NE		0.22	21.02	(1.74, 21.02)	0.45
≥3	17.73	(13.29, 33.75)		13.03	(7.83, NE)		7.37	(2.99, 11.32)	
**ECOG**									
0–1	**21.81**	**(16.32, 35.82)**	**0.005**	**13.03**	**(10.43, NE)**	**0.001**	9.34	(3.26, 36.81)	0.76
2–4	**8.82**	**(1.15, NE)**		**4.84**	**(0.13, 4.84)**		9.05	(NE)	
**National ADI**									
Low ADI	21.25	(13.03, 33.75)	0.68	13.03	(7.07, NE)	1.00	11.32	(6.51, 36.81)	0.08
High ADI (>9)	24.38	(10.66, NE)		NE	(0.13, NE)		5.58	(1.74, 21.02)	

For patients treated with MIRV and T-DXd, the age cutoff used for analysis was 65 years, whereas for those treated with TV, an age cutoff of 55 years was applied.

Acronym: NE-not evaluable; MIRV- Mirvetuximab soravtansine; T-DXd- Trastuzumab deruxtecan; TV- Tisotumab vedotin; Area deprivation index (ADI).

**Table 3:** Median Progression Free Survival (months) for Gynecologic Antibody–Drug Conjugates by Age, Race, Ethnicity, Line ofTreatment, ECOG Performance Status, and Area Deprivation Index.

Variable	MIRV			T-DXd			TV		
	Median PFS	(95% CI)	p-value	Median PFS	(95% CI)	p-value	Median PFS	(95% CI)	p-value

Total	7.3	(5.5, 13.5)	–	6.5	(2.4, NE)	–	4.8	(1.4, NE)	–
**Age**									
<65/55	8.3	(5.2, 15.9)	0.50	11.6	(5.6, NE)	0.33	4.6	(1.4, NE)	0.37
≥55/65	21.3	(13.03, 33.75)		7.0	(0.6, NE)		6.7	(1.4, NE)	
**Race**									
White	6.6	(5.2, 11.2)	0.28	11.6	(5.6, NE)	0.24	4.6	(1.4, NE)	0.88
Non-White	14.44	(11.55, NE)		6.7	(1.4, NE)		4.8	(1.4, NE)	
**Ethnicity**									
Hispanic	5.1	(1.6, NE)	0.40	7.5	(7.0, NE)	0.89	NE	(1.4, NE)	0.77
Non-Hispanic	8.2	(5.5, 13.9)		7.1	(5.6, NE)		4.8	(1.4, NE)	
Other	NE	(1.4, NE)		NE	(0.6, NE)		–		
**Treatment Line**									
1–2	16.8	(5.5, NE)	**0.04**	NE	(5.8, NE)	0.74	4.8	(1.4, NE)	0.53
≥3	6.6	(4.7, 9.4)		7.1	(4.3, NE)		3.2	(1.4, NE)	
**ECOG**									
0–1	7.3	(5.5, 13.5)	0.42	8.1	(5.6, NE)	0.36	4.8	(1.4, NE)	0.40
2–4	4.9	(0.7, NE)		NE	(3.3, NE)		3.2	(NE)	
**National ADI**									
Low ADI	8.2	(4.6, NE)	0.32	7.1	(5.6, NE)	0.31	NE	(1.4, NE)	0.05
High ADI (>9)	6.6	(4.8, NE)		NE	(2.8, NE)		3.2	(1.4, NE)	

Patients treated with MIRV and T-DXd, the age cutoff used for analysis was 65 years, whereas for those treated with TV, an age cutoff of 55 years was applied.

Acronym: NE-not evaluable; MIRV- Mirvetuximab soravtansine; T-DXd- Trastuzumab deruxtecan; TV- Tisotumab vedotin; Area deprivation index (ADI).

## HIGHLIGHTS

- Real-world ADC outcomes evaluated in underrepresented gynecologic cancer patients.
- No independent survival differences by race, ethnicity, or age after adjustment.
- ECOG and ethnicity were associated with OS in univariate analysis.
- Treatment line was associated with PFS outcomes with mirvetuximab.
- ADC effectiveness was consistent with results from pivotal clinical trials.

## Appendix A. Supplementary data

Supplementary data to this article can be found online at https://doi.org/10.1016/j.ygyno.2026.04.011.
