# Supplementary material for: Are patient factors associated with real-world antibody–drug conjugate outcomes in gynecologic cancers?
Source: Gynecol Oncol. Author manuscript; Available in PMC 2026 Oct 7. (PMC13644882; doi:10.1016/j.ygyno.2026.04.011)
Supplement: Supplementary file 1 [file NIHMS2205822-supplement-Supplementary_Meterial.docx]

**Supplemental Tables**

**Table S1.** Study population treated with doublet/triplet regimens

| **ADC Treatment Regimen N=24** | **N, (%)** |
| --- | --- |
| **Mirvetuximab soravtansine** |  |
| MIRV+ Bevacizumab | 14 (13.3) |
| MIRV + Carboplatin | 7 (6.7) |
| MIRV + Gemcitabine | 1 (1.0) |
| **Trastuzumab deruxtecan** |  |
| T-DXd + Nivolumab | 1 (2.9) |
| T-DXd +Pembrolizumab + Bevacizumab | 1 (2.9) |

Acronym: ADC-Antibody drug conjugate; MIRV- Mirvetuximab soravtansine; T-DXd- Trastuzumab deruxtecan; TV- Tisotumab vedotin; ADI- Area deprivation index

**Table S2.** Immunohistochemistry and Genomic Biomarker Profile of the Study Cohort

| Variable n, % | **Total Population***  (n=142) | **Mirvetuximab soravtansine** (n=105) | **Trastuzumab deruxtecan** (n=34) | **Tisotumab vedotin**  (n=16) |
| --- | --- | --- | --- | --- |
| BRCA mutation* |  | 24 (22.9) |  |  |
| BRCA1/2 germline pathogenic variant |  | 17 (16.2) |  |  |
| HR Deficient |  | 50 (47.6) |  |  |
| HR Proficient |  | 48 (45.7) |  |  |
| HR Unknown |  | 7 (6.7) |  |  |
| **HER2 expression level** |  |  |  |  |
| 0 | 41 (28.9) | 37 (35.2) | 0 | 4 (25.0) |
| 1+ | 24 (16.9) | 22 (21.0) | 6 (17.6) | 2 (12.5) |
| 2+ | 20 (14.1) | 10 (9.5) | 18 (52.9) | 2 (12.5) |
| 3+ | 6 (4.2) | 3 (2.9) | 5 (14.7) | 0 |
| Unknown | 38 (26.8) | 26 (24.8) | 5 (14.7) | 8 (50.0) |
| Amplification** | 13 (9.2) | 7 (6.7) | 8 (23.5) | 0 |
| **FRα expression level** |  |  |  |  |
| ≥75% |  | 84 (80.0) |  |  |
| <75% |  | 21 (20.0) |  |  |

*BRCA somatic and germline mutations **Her2 amplification and IHC positivity were not mutually exclusive

**Supplemental Figures**

**Figure S1.** Kaplan–Meier analysis of Median Overall Survival of Ovarian Cancer Patients treated with Mirvetuximab sorvatansine by **(a)** Age at ADC initiation, **(b)** Race, **(c)** Ethnicity, **(d)** Line of Treatment and **(e)** ECOG Performance Status.

(a) (b)

(c )	(d)

(e)

***Figure S1. Overall Survival by Age, Race, Ethnicity, Line of treatment and ECOG Performance status in Patients Receiving Mirvetuximab Soravtansine* a.** Median OS was 21.3 months for patients aged ≥65 years (95% CI 13.0–33.8) and 21.8 months for those <65 years (95% CI 12.4–NE; *p = 0.84*). **b.** By race, median OS was 21.3 months (95% CI 15.5–NE) in White patients versus 14.4 months (95% CI 11.5–NE) in non-White patients (*p = 0.81*). **c.** By ethnicity, median OS was 11.8 months (95% CI 5.2–43.2) for Hispanic, 24.4 months (95% CI 16.9–35.8) for non-Hispanic, and 3.4 months (95% CI 1.4–11.5) for other ethnicities (*p < 0.0001*). Median OS was NE (95% CI 11.3-NE) for 1–2 lines and 17.7 months (95% 13.3-33.8) for ≥3 lines (log-rank *p = 0.42*). **b.** *Overall Survival by ECOG Performance Status in Patients Receiving MIRV.* Patients with ECOG 0–1 (*n = 93*) demonstrated significantly longer survival (median 21.8 months 95% CI 16.3-35.8) than those with ECOG ≥ 2 (*n = 6*, median 8.8 months 95% CI 1.2-NE); log-rank *p < 0.005*).

**Figure S2.** Kaplan–Meier analysis of Median Overall Survival of Gynecologic Cancer Patients treated with Trastuzumab deruxtecan by **(a)** Age at ADC initiation, **(b)** Race, **(c)** Ethnicity, **(d)** Line of Treatment and **(e)** ECOG Performance Status.

(a) (b)

(b) (d)

(e)

***Supplementary Figure S2. Overall Survival by Age, Race, Ethnicity, Line of treatment and ECOG performance status in Patients Receiving Trastuzumab Deruxtecan* a.** Median OS was 13.0 months (95% CI 7.8–NE) for patients ≥65 years and 11.3 months (95% CI 2.4–NE) for <65 years (*p = 0.31*). **b.** By race, median OS was 12.0 months (95% CI 7.1–NE) among White versus 13.0 months (95% CI 4.8–NE) among non-White patients (*p = 0.52*). **c.** By ethnicity, median OS was 14.2 months for Hispanic (95% CI NE), 12.0 months (95% CI 7.8–NE) for non-Hispanic, and NE for other groups (*p = 0.97*). **d.** Median OS was NE and 13.0 months, (95% CI 7.8-NE), respectively (*p = 0.22*). **e.** Patients with ECOG 0–1 (*n = 31*) had significantly longer median OS 13.0 months (95% CI 10.4-NE) compared with those with ECOG ≥ 2 (*n = 3*, median 4.8 months, 95% CI 0.1-4.8); *p = 0.001*).

**Figure S3.** Kaplan–Meier analysis of Median Overall Survival of Cervical Cancer Patients treated with Tisotumab vedotin by **(a)** Age at ADC initiation, **(b)** Race, and **(c)** Ethnicity, **(d)** Line of Treatment and **(e)** ECOG Performance Status.

(a) (b)

(b) (d)

(e)

***Supplementary Figure 3S. Overall Survival by Age, Race, and Ethnicity in Patients Receiving Tisotumab Vedotin*** **a.** Median OS was 9.0 months (95% CI 3.3–36.8) for patients ≥55 years and 7.4 months (95% CI 1.7–21.0) for <55 years (*p = 0.36*). **b.** By race, median OS was 21.0 months (95% CI 3.3–36.8) in White and 5.6 months (95% CI 1.7–11.3) in non-White patients (*p = 0.13*). **c.** By ethnicity, median OS was 6.5 months (95% CI 1.7–11.3) among Hispanic and 9.0 months (95% CI 3.3–36.8) among non-Hispanic patients (*p = 0.26)* **d.** Median OS was 21.0 months (95% CI 1.7-21.0) for 1–2 lines (*n = 9*) vs 7.4 months (95% CI 3.0-11.3) for ≥3 lines (*n = 7*; *p = 0.45*). **e.** Kaplan–Meier analysis by ECOG status showing no statistically significant difference in OS (ECOG 0–1 *n = 15*, median 9.3 months (95% CI 3.2-36.8); ECOG ≥ 2 *n = 1*, median 9.0 months, 95% CI NE; *p = 0.7565*).

**Figure S4.** Progression Free Survival by Underrepresented subgroups of Gynecologic Cancer patients treated with Antibody Drug Conjugates

***Figure S4. Forest plots depicting median progression-free survival (PFS) and 95% confidence intervals (CI) across patient subgroups for mirvetuximab soravtansine (MIRV), trastuzumab deruxtecan (T-DXd), and tisotumab vedotin (TV) in patients with advanced gynecologic malignancies*.** Point estimates represent median PFS in months; horizontal lines represent 95% confidence intervals with error bars indicating bounds. Arrows indicate confidence intervals extending beyond the upper bound (not estimable, NE). The vertical dashed line represents the overall median PFS for each treatment. Age cutoffs were <65 vs ≥65 years for MIRV and T-DXd, and <55 vs ≥55 years for TV. Subgroup analyses include age, race (white vs non-white), ethnicity (Hispanic, non-Hispanic, other), line of treatment (1-2 vs ≥3), ECOG performance status (0-1 vs 2-4), and National Area Deprivation Index (Low ADI vs High ADI >9). NE indicates confidence interval bound not estimable due to insufficient events or censoring. ADI-Area Deprivation Index; CI-confidence interval; ECOG-Eastern Cooperative Oncology Group; NE-not estimable; PFS-progression-free survival.
